# Detection of Pulmonary Infectious Pathogens From Lung Biopsy Tissues by Metagenomic Next-Generation Sequencing

**DOI:** 10.3389/fcimb.2018.00205

**Published:** 2018-06-25

**Authors:** Henan Li, Hua Gao, Han Meng, Qi Wang, Shuguang Li, Hongbin Chen, Yongjun Li, Hui Wang

**Affiliations:** ^1^Department of Clinical Laboratory, Peking University People's Hospital, Beijing, China; ^2^BGI Genomics, Beijing Genomics Institute-Shenzhen, Shenzhen, China

**Keywords:** metagenomic next-generation sequencing (mNGS), pulmonary infection, lung biopsy tissues, data management, clinical diagnosis

## Abstract

Metagenomic next-generation sequencing (mNGS) is a comprehensive approach for sequence-based identification of pathogenic microbes. However, reports on the use of mNGS in pulmonary infection applied to lung biopsy tissues remain scarce. In this study, we applied mNGS to detect the presence of pathogenic microbes in lung biopsy tissues from 20 patients with pulmonary disorders indicating possible infection. We applied a new data management for identifying pathogen species based on mNGS data. We determined the thresholds for the unique reads and relative abundance required to identify the infectious pathogens. Potential pathogens of pulmonary infections in 15 patients were identified by mNGS. The comparison between mNGS and culture method resulted that the sensitivity and specificity were 100.0% (95% CI: 31.0–100.0%) and 76.5% (95% CI: 49.8–92.2%) for bacteria, 57.1% (95% CI: 20.2–88.2%) and 61.5% (95% CI: 32.2–84.9%) for fungi. The positive predictive value (PPV) (42.9% for bacteria, 44.4% for fungi) was much lower than negative predictive value (NPV) (100% for bacteria, 72.7% for fungi) in mNGS vs. culture method. The mNGS showed the highest specificity (100.0 and 94.1%) and PPV (100.0 and 75.0%) in the evaluation of fungi and MTBC respectively, when compared with histopathology method. The study indicated that mNGS of lung biopsy tissues can be used to detect the presence (or absence) of pulmonary pathogens in patients, with potential benefits in speed and sensitivity. However, accurate data management and interpretation of mNGS are required, and should be combined with observations of clinical manifestations and conventional laboratory-based diagnostic methods.

## Introduction

Pulmonary infection is a leading cause of death and morbidity worldwide (Magill et al., 2014). However, its diagnosis is challenging due to the multitude of possible pathogens. Hundreds of pathogens have been associated with pulmonary infections, including bacterial, viral, or fungal pathogens (Renaud and Campbell, 2011; Ruppé et al., 2016; De La Cruz and Silveira, 2017). In immunocompromised patients, virtually any bacteria or fungus can be considered as a potential pneumonia-causing pathogen.

A rapid microbiological diagnosis of pulmonary infections facilitates the timely application of antimicrobial therapy. Smear by microscopy and culture are the conventional microbiological methods used to identify pathogens, but both methods are relatively insensitive and culture is time-consuming. Histopathological diagnosis is the diagnostic gold standard of invasive fungal infections. However, it requires time and is not pathogen-specific. Rapid advances in sequencing technology and bioinformatics have made metagenomic next-generation sequencing (mNGS) a fertile area for the development of clinical diagnostics (Hilton et al., 2016; Salzberg et al., 2016; Somasekar et al., 2017). Culture-independent screening for pathogens with mNGS only needs a small amount of DNA directly taken from the sample, and a bioinformatics tool, which identifies pathogens by linking sequencing reads to an accurate reference genome (or marker) database (Forbes et al., 2017). Eventually, if the sequence depth is sufficient enough, the antibiotic susceptibility of pathogens can be inferred. Recent work has highlighted the current interest in using mNGS for the identification and antibiotic susceptibility testing of pathogens in the diagnosis of viral acute encephalitis (Naccache et al., 2015), infective endocarditis (Fukui et al., 2015), and bacterial meningitis (Salzberg et al., 2016). However, reports on the use of mNGS in pulmonary infection applied to lung biopsy tissues remain scarce.

In particular, we applied mNGS on 20 computer tomography (CT)-guided puncture lung biopsy tissues collected from patients with suspected pulmonary infections. After mNGS, we analyzed the relative abundance, coverage, depth, and unique reads of microbial sequences mapping to fungal and bacterial reference genomes from NCBI. The results obtained by mNGS were compared with those from conventional laboratory-based diagnostic methods. Our results indicated that mNGS provided a major new opportunity to investigate the pathogens of pulmonary infections.

## Materials and methods

### Ethics statement

Application for ethical review was submitted to the Ethical Review Committee of Peking University People's Hospital (Reference 2017PHB075). The study was considered exempt from ethical review as it was a retrospective study and patients were anonymized.

### Specimen collection and processing

CT-guided puncture lung biopsy tissues with suspected pulmonary infections were collected from Peking University People's Hospital according to standard procedures. A total of 20 samples collected between February 2016 and October 2017 were investigated in this study. Of these, 12 samples were culture negative, and eight samples were both smear and culture positive. The lung biopsies were separately sent to clinical microbiology and histopathology laboratories within 2 h for analyses. The histopathology laboratory used standard methods for processing clinical samples. In the clinical microbiology laboratory, tissues were homogenized in 2 mL brain heart infusion broth in a glass grinder, and used for smear and culture. Gram stain, KOH test, and Ziehl-Neelsen stain were used to identify bacteria, fungi and *Mycobacterium tuberculosis* complex (MTBC) by smear microscopy. Homogenized samples were inoculated onto blood agar, eosin methylene blue agar and chocolate agar (Oxoid, Basingstoke, Hampshire, UK) at 35°C to isolate bacteria for up to 5 days. Sabouraud dextrose agar supplemented with and without chloramphenicol (Oxoid, Basingstoke, Hampshire, UK) were used to isolate fungi at 28 and 35°C for up to 5 days, respectively. All cultured microorganisms were identified using the Vitek 2 automated system (bioMérieux, Marcy-l'Etoile, France). Filamentous fungi were identified according to colony morphology and smear results. The remaining or left over tissue homogenates were stripped of patient identification details and stored at −70°C for mNGS.

### Isolation of genomic DNA, library preparation, and mNGS

DNA of samples were extracted directly from the tissue homogenates with a TIANamp Micro DNA Kit (TIANGEN BIOTECH). The extracted DNA was fragmented ultrasonically to yield 200–500 bp fragments. After fragmentation by sonication or fragmentase, the DNA fragments were underwent end-repairing, phosphorylation and A-tailing reactions. BGISEQ-500 platform-specific adaptors were ligated to the A-tailed fragments, and the ligated fragments were purified, and then amplified using PCR. Finally, circularization was performed to generate single stranded DNA circles. After quantitation and qualification, the libraries were sequenced. BGI performed the DNA nanoball preparation and whole genome sequencing using the circular single stranded libraries as a template for rolling circle amplification to form DNA nanoballs. The DNA nanoballs were loaded onto a sequencing flow cell and then processed for 50 bp single end sequencing on the BGISEQ-500 platform (Fang et al., 2017). Samples were extracted in batches, with a negative control of whole blood sample from healthy donors prepared alongside each batch using this same protocol.

### Bioinformatics analyses

High quality sequencing data were generated by removing low quality reads, adapter contamination, and duplicated reads, as well as those shorter than 35 bp. Human sequence data were identified by mapping to a human reference (hg19) using Burrows-Wheeler Aligner software (Li and Durbin, 2009) and excluded. The nonhuman sequence reads from each sample were deposited at Genome Sequence Archive of Beijing Institute of Genomics, Chinese Academy of Sciences (gsa.big.ac.cn) under accession number PRJCA000880. The remaining sequence data were aligned to the current bacterial, virus, fungal, and protozoan databases (NCBI; ftp://ftp.ncbi.nlm.nih.gov/genomes). The database used for this study contained 1,428 bacterial species, 1,130 viral species, 73 fungal species, 48 parasites, four species of the MTBC (*M. tuberculosis, Mycobacterium canettii, Mycobacterium africanum*, and *Mycobacterium bovis*), and 40 mycoplasma/chlamydia related to human diseases. Unique reads were defined as reads whose alignment length was higher than 90%, identity with reference sequence higher than 95%, and ratio of suboptimal to optimal alignment score lower than 0.8. The infectious bacteria or fungi were determined if it met any of the following thresholds: (i) >30% relative abundance at the genus level in bacteria or fungi; (ii) culture and/or histopathological examination positive and at least 50 unique reads from a single species of bacteria or fungi; (iii) at least one unique read from MTBC.

### Statistical analyses

In accordance with the extracted data, 2 × 2 contingency tables were derived to determine sensitivity, specificity, positive predictive value (PPV), negative predictive value (NPV). All statistics have reported as absolute values with their 95% confidence interval (95% CI). Sensitivity and specificity were calculated on the basis of the formulas TP (true positive)/TP + FN (false negative) and TN (true negative)/TN + FP (false positive), respectively. PPV is expressed by the TP/TP+FP ratio, while NPV from the TN/TN+FN.

## Results

### Patient demographics and basic sequencing information

**Figure 1:**
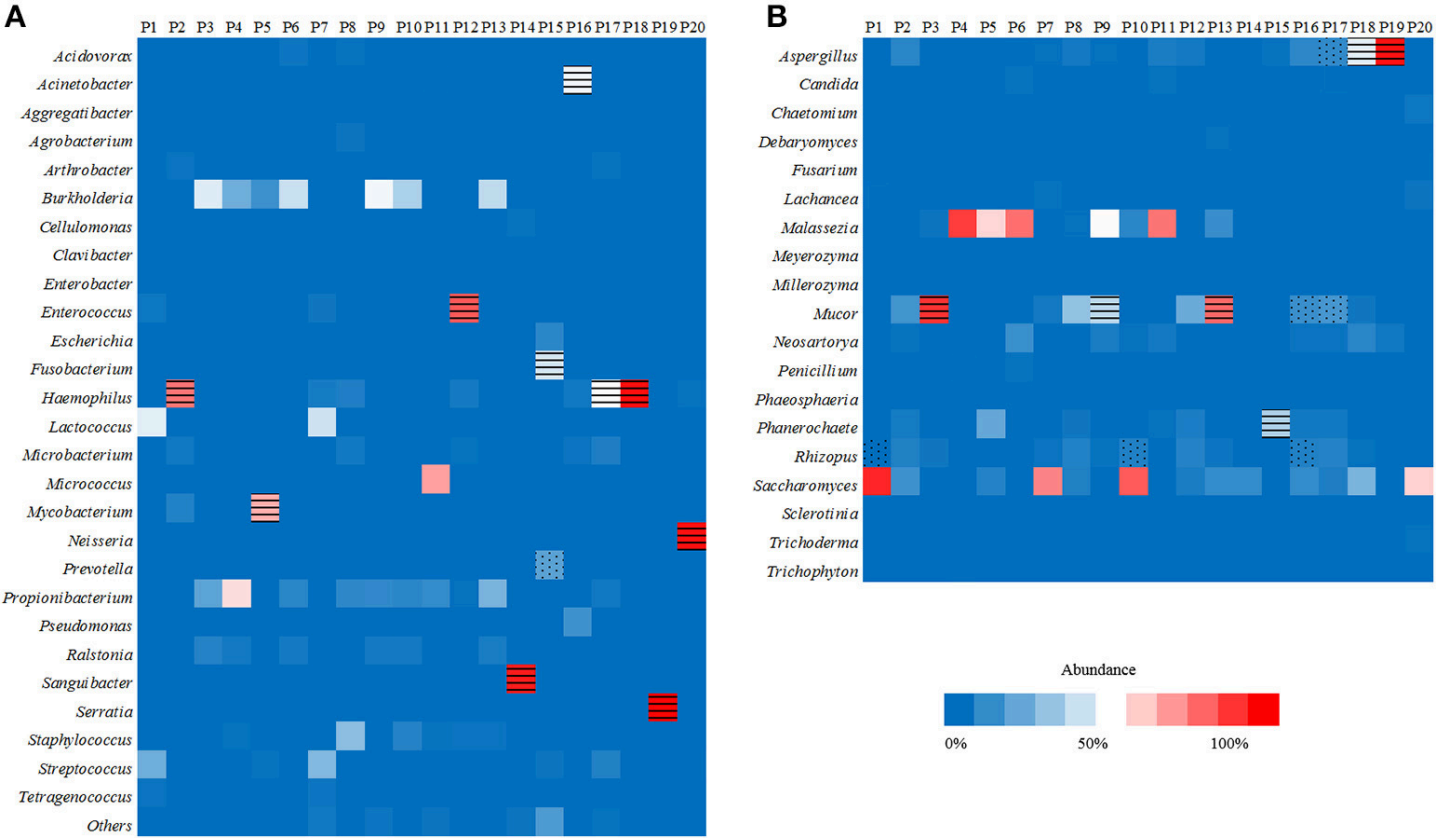
Heat maps reporting the relative abundance at the genus level of the 10 most abundant bacterial species **(A)** and fungal species **(B)** identified by metagenomic next-generation sequencing (mNGS) in 20 lung biopsy tissues. The infectious bacteria or fungi were marked with black horizontal lines if >30% relative abundance at the genus level, or marked with black scatter spots if included at least 50 unique reads from culture and/or histopathological examination positive bacteria or fungi.

**Figure 2:**
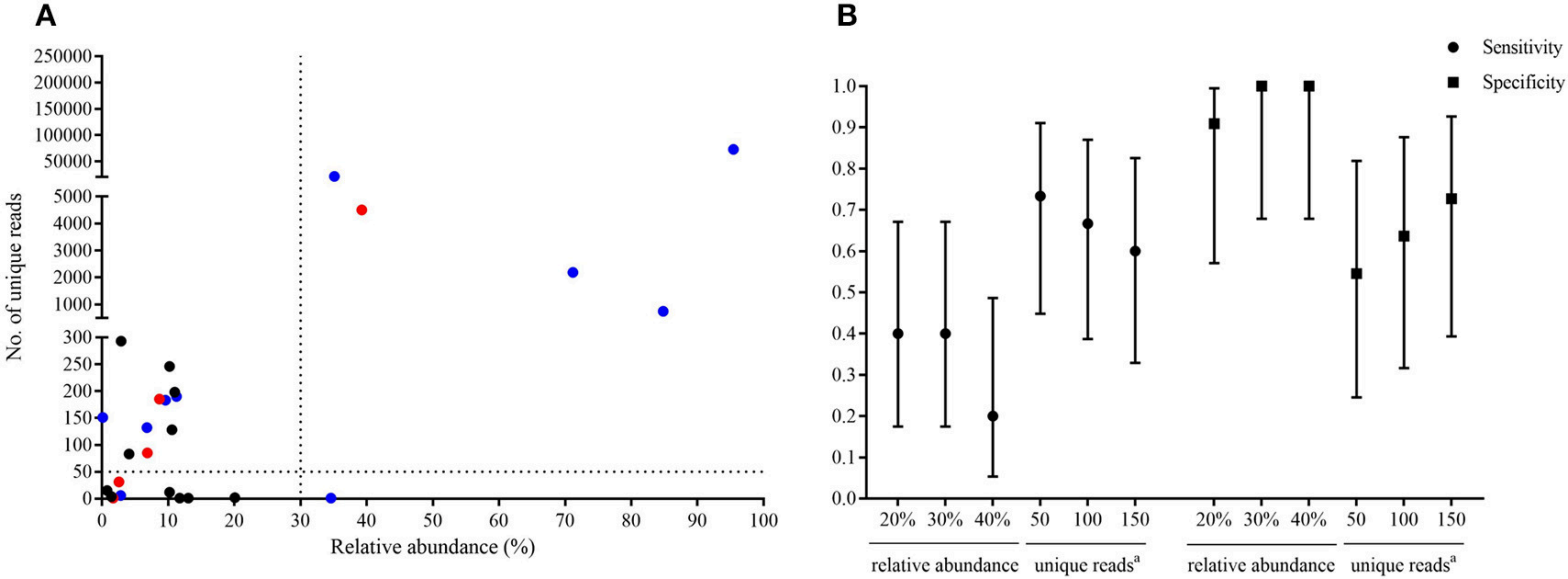
Optimal thresholds for infectious fungi identification by metagenomic next-generation sequencing (mNGS). The fungal species which contained the highest relative abundance and maximum number of unique reads in each sample were selected, and the relative abundance and unique reads were shown in **(A)**. The red dots indicated fungi identified by mNGS in samples which were consistent with culture results at the genus or species level. Blue dots indicated species identified by mNGS in samples which showed fungal hypha by histopathological examination. Black dots indicated species identified by mNGS in samples which had negative results of fungi by conventional culture and/or histopathology methods. Sensitivity and specificity with 95% confidence intervals were calculated under different threshold **(B)**.

Of the 20 CT-guided puncture lung biopsy tissues processed using mNGS analysis, eight samples were culture positive, and fungal components or acid-fast bacteria were identified in 13 samples by histopathological examination. Potential pathogens of pulmonary infections in 15 patients were identified by mNGS (Table 1). The sequenced biopsies generated between 0.7 and 69 million reads per sample (Supplemental Table S1). The mNGS provide a wide range of microbial profiles, which were difficult to interpret. The microbial species were ranked by relative abundance (Figure 1) and the threshold was determined to identify the presence of true infectious pathogens. *Propionibacterium acnes, Micrococcus luteus, Malassezia globosa, Lactococcus lactis*, and *Saccharomyces*, which were known normal flora of the skin or respiratory tract, were not interpreted as pathogens (Aas et al., 2005; Byrd et al., 2018). Optimal thresholds for determining if samples contained low-level contamination or true infection were determined by numerical optimization (Figure 2). We determined the 30% relative abundance as the final threshold that maximized the sensitivity (40.0%, 95% CI: 17.5–67.1%) and specificity (100.0%, 95% CI: 67.9–100%) of mNGS for fungal infection. For culture and/or histopathology positive samples, 50 unique reads threshold from a single species of fungi was selected to maximized the sensitivity (73.3%, 95% CI: 44.8–91.1%) and reduce missed diagnosis. We determined the same thresholds for the relative abundance and unique number of bacteria required to identify the infectious bacteria according to fungi thresholds, and confirmed our findings in two bacterial culture positive samples. Meanwhile, more fastidious and anaerobic bacteria were identified by mNGS than the conventional culture method (Table 2).

**Table 1:** Pulmonary disorders patients for metagenomic next-generation sequencing and conventional laboratory-based diagnostic testing.

**Patient ID**	**Sex**	**Age/yr**	**Underlying disease**	**Pulmonary disorders**	**Immunocompromised**	**Smear results**	**Culture results**	**Pathology results**	**Clinical nucleic acid testing results**	**mNGS based diagnosis**
P1	Male	26	AML; allo-HSCT	Fungal infections	Yes	Fungal nonseptatehypha (90 degrees)	Negative	Fungal hypha (probable *Zygomycetes*)	ND	Fungal infection
P2	Female	43	Uterine leiomyoma (postoperative)	Pulmonary occupying lesion	No	Fungal hypha	Negative	Several acid-fast bacteria	MTBC positive by quantitative real-time PCR	MTBC infection, bacterial infection
P3	Female	54	ALL	Pulmonary infection	Yes	Fungal nonseptatehypha (90 degrees)	Negative	Fungal hypha	ND	Fungal infection
P4	Male	51	Type 2 diabetes mellitus	Pulmonary tuberculosis	No	Acid-fast bacteria	Negative	Single suspicious acid-fast bacterium	MTBC positive by GeneXpert	MTBC infection
P5	Male	60	Type 2 diabetes mellitus; excision of thyroid adenoma	Pulmonary tuberculosis	No	Acid-fast bacteria	Negative	Acid-fast bacteria	ND	MTBC infection
P6	Female	49	None	Lung cancer (adenocarcinoma)	No	Gram-negative bacilli	Negative	Adenocarcinoma	MTBC negative by GeneXpert	Noninfectious
P7	Female	61	None	Pulmonary inflammatory lesions; Pulmonary occupying lesion	No	Negative	Negative	Chronic nonspecific inflammatory	ND	Noninfectious
P8	Female	40	None	Pulmonary occupying lesion, pulmonary infection	No	Negative	Negative	Scattered lymphocyte infiltration	ND	Noninfectious
P9	Female	24	ALL; allo-HSCT	Pneumonia (coinfection, fungi confirmed)	Yes	Negative	Negative	Fungal hypha	ND	Fungal infection
P10	Male	49	AML; allo-HSCT	Fungal infections	Yes	Negative	Negative	Fungal hypha	ND	Fungal infection
P11	Male	58	Chronic myelogenous leukemia; COPD	Lung squamous cell carcinoma	Yes	Negative	Negative	Probable squamous cell carcinoma	ND	Noninfectious
P12	Male	66	COPD	Lung cancer	No	Negative	Negative	Probable a malignant tumor with epithelioid differentiation	MTBC negative by GeneXpert	Noninfectious
P13	Female	39	AML; allo-HSCT	Fungal infections	Yes	Negative	*Rhizopus*	Fungal hypha	MTBC negative by quantitative real-time PCR	Fungal infection
P14	Female	43	Chronic hepatitis B virus hepatitis	Pulmonary tuberculosis (high probability)	No	Negative	*Aspergillus fumigatus*	Scattered lymphocyte infiltration; granuloma formation	MTBC positive by GeneXpert	MTBC infection
P15	Male	54	Hypertension	Pulmonary inflammatory lesions	No	Fungal hypha	*Aspergillus fumigatus, Prevotella intermedia*	Chronic nonspecific suppurative inflammation	ND	Bacterial infection
P16	Male	29	AML; allo-HSCT	Lung infection (fungi, bacteria)	Yes	Fungal hypha	*Rhizopus*	Fungal components (probable *Zygomycetes*)	ND	Fungal infection, bacterial infection
P17	Female	73	Coronary atherosclerosis	Fungal infections	No	Fungal hypha	*Aspergillus fumigatus*	Fungal components	ND	Fungal infection, bacterial infection
P18	Female	39	None	Fungal infection	No	Fungal hypha	*Aspergillus fumigatus, Staphylococcus haemolyticus*	Fungal components	ND	Fungal infection, bacterial infection
P19	Male	46	None	Fungal infections	No	Gram-negative bacilli	*Serratia marcescens*	Fungal hypha	ND	Fungal infection, bacterial infection
P20	Male	6	ALL; post-right lower lung resection	Pulmonary infection	Yes	Fungal septatehypha	*Penicillium*	Fungal hypha (probable filamentous fungi except for *Aspergillus* and *Zygomycetes*)	MTBC negative by quantitative real-time PCR	Bacterial infection

*AML, Acute myeloid leukemia; ALL, Acute lymphocytic leukemia; Allo-HSCT, allogeneic hematopoietic stem cell transplantation; COPD, chronic obstructive pulmonary disease; MTBC, Mycobacterium tuberculosis complex; PCR, polymerase chain reaction; ND, no data*.

**Table 2:** Metagenomic next-generation sequencing results of 20 lung biopsy samples.

**Patient ID**	**Culture results**	**Metagenomic results**				
		**Species**	**No. of unique reads**	**Coverage, %**	**Depth**	**mNGS based diagnosis**
P1	Negative	*Rhizopus microsporus*^a^	151	0.0425	1.31	Fungal infection
P2	Negative	*Mycobacterium canettii* (MTBC)	1	0.038	1	MTBC infection, bacterial infection
	Negative	*Haemophilus influenzae*	1,500	3.1	1	
P3	Negative	*Mucor racemosus*	743	0.21	1	Fungal infection
P4	Negative	*Mycobacterium canettii* (MTBC)	5	0.021	1	MTBC infection
P5	Negative	*Mycobacterium tuberculosis* (MTBC)	5	0.9	1	MTBC infection
P6	Negative	Negative	*/*	*/*	*/*	Noninfectious
P7	Negative	Negative	*/*	*/*	*/*	Noninfectious
P8	Negative	Negative	*/*	*/*	*/*	Noninfectious
P9	Negative	*Mucor racemosus*	21,548	0.14	1	Fungal infection
P10	Negative	*Rhizopus oryzae*^a^	132	0.073	1	Fungal infection
P11	Negative	Negative	*/*	*/*	*/*	Noninfectious
P12	Negative	*Enterococcus faecalis*^b^	453	3.5	1	Noninfectious
P13		*Mucor racemosus*	2,187	0.27	1	Fungal infection
	*Rhizopus*	*Rhizopus oryzae*^ad^	31	0.011	1	
P14	*Aspergillus fumigatus*	*Sanguibacter keddieii*^b^	1,425,089	50	75	
		*Mycobacterium tuberculosis* (MTBC)	0 (23 from MTBC)	0.027	1.5	MTBC infection
P15		*Phanerochaete chrysosporium*^b^	1	0.011	1.3	
	*Aspergillus fumigatus*	*Aspergillus fumigatus*^d^	1	0.00041	1.7	
		*Fusobacterium nucleatum*^c^	69	0.2478	1	Bacterial infection
	*Prevotella intermedia*	*Prevotella intermedia*^a^	57	0.1153	1	
P16	*Rhizopus*	*Rhizopus microsporus*^a^	85	0.0106	9	Fungal infection, bacterial infection
		*Mucor indicus*^a^	183	0.0036	36.35	
		*Acinetobacter lwoffii*	660	1.6	1.1	
P17	*Aspergillus fumigatus*	*Aspergillus fumigatus*^a^	185	0.0343	2.91	Fungal infection, bacterial infection
		*Mucor indicus*^a^	190	0.0033	39.42	
		*Haemophilus influenzae*	242	0.66	1	
P18	*Aspergillus fumigatus*,	*Aspergillus fumigatus*	4,508	1.1149	1.01	Fungal infection, bacterial infection
	*Staphylococcus haemolyticus*	*Haemophilus influenzae*	378,078	84	12	
P19	*Serratia marcescens*	*Serratia marcescens*	227,748	82	8.1	Fungal infection, bacterial infection
		*Aspergillus fumigatus*	73,043	32	1.2	
P20	*Penicillium*	*Chaetomium globosum*^abd^	6	0.0036	1.1	
		*Neisseria meningitidis*	4,247	15	3.9	Bacterial infection

a *Inferred pathogen combined with culture and/or histopathological examination results*.

b *Rare pathogen in pulmonary infection, which was not interpreted as pathogen*.

c *Obligate anaerobe*.

d *Below the thresholds*.

MTBC include *M. tuberculosis, M. canettii, M. africanum*, and *M. bovis* in the mNGS database. The genome sequence of the four *Mycobacterium* showed high genomic similarity, so there are few species specific unique reads. For MTBC infection, we suggested focus on matching the number of unique reads in MTBC, rather than unique reads in species. Since the clinical MTBC nucleic acid testing result was positive when only one unique read of MTBC detected by mNGS, we determined the threshold of MTBC infection as one unique read. The number of unique reads of the identified pathogens by mNGS ranged from 1 to 1,425,089. The coverage of identified pathogens ranged from 0.0033 to 84% with a depth value of 1–75, respectively.

### Identification of pathogen species in culture negative samples by mNGS

Of 12 samples which had negative culture results, mNGS analysis produced negative or nonspecific findings in five samples (P6~P8, P11, and P12) and positive pathogen identification in seven samples (P1~P5, P9, and P10; Table 1). Two samples (P4 and P5) were positive for acid-fast bacteria identification by smearing, and mNGS data confirmed laboratory-based diagnostic testing results. In P5, mNGS analysis identified the MTBC as the most abundant bacteria with 65.71% relative abundance in bacteria and 1,627 unique reads at the genus level. In P4, the number of unique reads of MTBC was 205. GeneXpert MTBC testing was consistent with mNGS result in lung biopsy tissues. Quantitative real-time PCR assay also confirmed positive amplification for MTBC-specific genomic DNA in bronchoalveolar lavage of these patient. Among the three samples which were positive for fungal hypha identification by smearing, fungal species were identified in two samples (P1 and P3) by mNGS (Table 2). Two smear negative samples (P9 and P10) were also positive for fungi by mNGS. Among these samples, *Mucor racemosus* conformed to the threshold and was considered as a possible infectious pathogen in P3 and P9. *Rhizopus microspores* (P1) and *Rhizopus oryzae* (P10), which were consistent with histopathological descriptions, were also interpreted as infectious pathogens. Both *Rhizopus* and *Mucor* belong to *Mucoraceae*, which are difficult to test in a routine microbiology laboratory. In sample P12 from a lung cancer patient, analyses at the species level detected *Enterococcus faecalis* by mNGS, which was not detected by culture. However, *E. faecalis* was not interpreted as pathogen because it was uncommon in pulmonary infection of immunocompetent patient.

Conflicting results between mNGS and conventional smear method were identified in two samples. Other laboratory-based diagnostic testing results were listed in Table 3. In P2, fungi identified by smearing were not detected by mNGS at the predetermined thresholds. A fraction of bacterial reads were assigned to *Haemophilus influenzae* and MTBC by mNGS. Several acid-fast bacteria were identified by histopathological examination in P2, which was consistent with mNGS result. MTBC positive result was also confirmed by quantitative real-time PCR. In P6, no dominant abundant bacteria species was identified by mNGS, which contained Gram negative bacilli according to the smear result. *Malassezia globosa* was dominant over other fungal species in this sample, but was not interpreted as a pathogen because it was known to normal flora of human skin (Byrd et al., 2018). We speculated P6 was noninfectious, which agreed with the clinical diagnosis (lung cancer) and the levels of serological infection markers procalcitonin (PCT) and C-reactive protein (CRP). The possible reasons for not detecting these pathogens by mNGS might be explained by predetermined thresholds or ambiguous smear results.

**Table 3:** The detail information of samples with conflicting results.

**Patient ID**	**Smear results**	**Culture results**	**Histopathology results**	**Potential pathogens by mNGS**	**Other laboratory-based diagnostic testing results**	**Clinical diagnosis of pulmonary disorders**
P2	Fungal hypha	Negative	Several acid-fast bacteria	MTBC *Haemophilus influenzae*	MTBC positive by quantitative real-time PCR WBC 14.80 × 10^∧^9/L (↑)	Pulmonary occupying lesion
P6	Gram-negative bacilli	Negative	Adenocarcinoma	Negative	MTBC negative by GeneXpert CRP 1.5mg/L PCT < 0.02ng/ml	Lung cancer (adenocarcinoma)
P14	Negative	*Aspergillus fumigatus*	Scattered lymphocyte infiltration; granuloma formation	MTBC	MTBC positive by GeneXpert	Pulmonary tuberculosis (high probability)
P20	Fungal septatehypha	*Penicillium*	Fungal hypha (probable filamentous fungi except for *Aspergillus* and *Zygomycetes*)	*Neisseria meningitidis*	WBC 11.06 × 10^∧^9/L (↑) CRP 16.62 mg/L (↑) MTBC negative by quantitative real-time PCR	Pulmonary infection

*MTBC, Mycobacterium tuberculosis complex; PCR, polymerase chain reaction; WBC, white blood cell count; CRP, C-reactive protein; PCT, procalcitonin*.

*Up arrow (↑) indicated that the result was higher than normal reference range*.

### Identification of pathogen species in culture positive samples by mNGS

The results from the mNGS analysis were consistent with three fungi culture results and two bacteria culture results at the species level in eight samples (P13–P20). Moreover, the mNGS identified more infectious pathogens than that by culture method (Table 2). The possible reasons for not detecting these pathogens in culture were that: *Fusobacterium nucleatum* is an obligate anaerobic bacterium, *H. influenzae* and *Neisseria meningitidis* are fastidious organisms, *Aspergillus fumigatus* requires longer culture period. The grind processing may affect the isolation of *Zygomycetes* (such as *Rhizopus* and *Mucor*). Moreover, pathogens isolation may not be possible especially if antibiotic treatment has already been initiated.

In P13, P15, P16, and P17, the fungi identified by culture were not the most abundant species in mNGS results. In P13, the fungal culture indicated *Rhizopus*, but *M. racemosus* had the highest abundance in fungi by mNGS (71.16%). *Rhizopus* was also identified in P13 with the low relative abundance (2.56%), which ranked fifth in species of fungi. The mNGS data showed the number of unique reads of *R*. *oryzae* was 31. *Mucor indicus* was identified in P16 and P17 as the most abundant species in fungi, but relative abundance were lower than 15%. In P15, *Phanero chaetechrysosporium* is a species of wood-rotting fungus and generally regarded as unable to induce diseases in human.

Conflicting data from mNGS and culture techniques were obtained in two samples (P14 and P20). Other laboratory-based diagnostic testing results were listed in Table 3. *Sanguibacter keddieii* was identified in P14 with 94.38% relative abundance in all reads assigned to bacteria by mNGS. Because pulmonary infection due to *Sanguibacter* is not common (Jones, 2010), *S. keddieii* was not interpreted as pathogen. Furthermore, mNGS identified the MTBC in P14, which was not detected by conventional smear methods. GeneXpert MTBC testing was consistent with mNGS result in P14. The possible reasons for not detecting these pathogens by conventional laboratory-based diagnostic methods could be explained by the fact that direct smear tests for MTBC have a low sensitivity. The P20 was a 6-year-old child, who had been admitted due to acute lymphocytic leukemia with central nervous system abnormalities. *Penicillium*, detected in fungal culture, was not specifically detected by mNGS. The mNGS identified *N. meningitidis* and *Saccharomyces cerevisiae* among the top most abundant species in bacteria and fungi respectively. *N. meningitidis* is a fastidious organism which lives in the nasopharynx, and *S. cerevisiae* is a known reagent contaminant. Although pulmonary infection due to *Neisseria* is uncommon (Hirai et al., 2016), physicians should consider *N. meningitidis* as possible pathogens in an immunocompromised patient. Histopathological examination indicated filamentous fungi except for *Aspergillus* and *Zygomycetes*. Further analysis of mNGS data showed *Chaetomium globosum* was ranked second by unique reads in species of fungi. However, *C. globosum* was not interpreted as a pathogen since it was only 6 unique reads.

### Comparison of conventional laboratory-based diagnostic methods and mNGS

**Figure 3:**
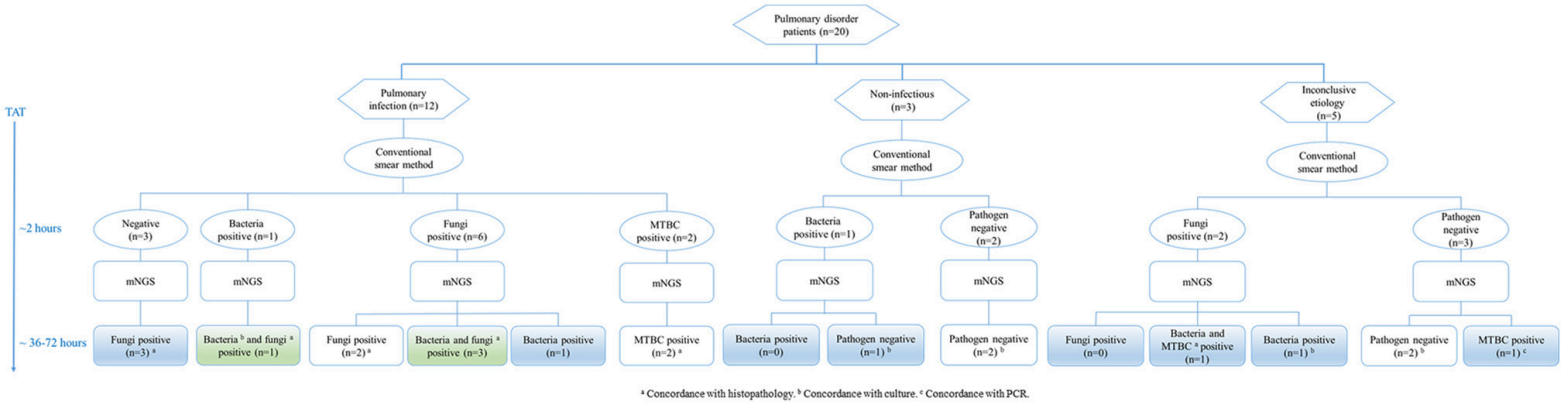
Testing of lung biopsy tissues from 20 patients with pulmonary disorders by conventional laboratory-based diagnostic methods and metagenomic next-generation sequencing (mNGS). MTBC, *Mycobacterium tuberculosis* complex; PCR, polymerase chain reaction; TAT, turn-around time.

Pulmonary disorder patients were classified into three groups according to the clinical diagnosis (Figure 3). In 12 patients diagnosed with pulmonary infections, two fungi positive and two MTBC positive samples detected by the conventional smear method were identified correctly by mNGS. Among six fungi positive samples detected by smearing, five were called fungi positive by mNGS. It's worth noting that three samples with a negative smear result were indicated as fungi positive by mNGS. Bacteria and fungi coinfections were identified in four cases, in which only one etiology was detected by smearing. Three samples with non-infectious etiologies were confirmed by mNGS. Importantly, one sample positive for bacteria positive by smearing was not detected by mNGS, which agreed with clinical diagnosis. The mNGS confirmed two of five inconclusive etiology cases, failing to detect fungi identified by smearing in two samples. Moreover, previously unrecognized MTBC infections and/or bacterial infections were found in two cases by mNGS.

The comparison of mNGS results with conventional microbiology methods (smear and culture) and histopathological method were shown in Table 4. The sensitivity and specificity of mNGS compared with smear method were 50.0% (95% CI: 2.7–97.3%) and 66.7% (95% CI: 41.2–85.6%) for bacteria, 62.5% (95% CI: 25.9–89.8%) and 66.7% (95% CI: 35.4–88.7%) for fungi, 100.0% (95% CI:19.8–100.0%) and 88.9% (95% CI: 63.9–98.1%) for MTBC, respectively. The comparison between mNGS and culture method resulted that the sensitivity and specificity were 100.0% (95% CI: 31.0–100.0%) and 76.5% (95% CI: 49.8–92.2%) for bacteria, 57.1% (95% CI: 20.2–88.2%) and 61.5% (95% CI: 32.2–84.9%) for fungi. The PPV (42.9% for bacteria, 44.4% for fungi) was much lower than NPV (100% for bacteria, 72.7% for fungi) in mNGS vs. culture method. The mNGS showed the highest specificity (100.0 and 94.1%) and PPV (100.0 and 75.0%) in the evaluation of fungi and MTBC, respectively, when compared with histopathology method.

**Table 4:** Diagnostic performance of metagenomic next-generation sequencing (mNGS) when compared with conventional laboratory-based diagnostic methods in the detection of bacteria, fungi, and *Mycobacterium tuberculosis* complex (MTBC).

**Pathogen**	**Conventional laboratory-based diagnostic method**	**mNGS**			
		**Sensitivity % (95% CI)**	**Specificity % (95% CI)**	**PPV % (95% CI)**	**NPV % (95% CI)**
Bacteria	Smear	50.0 (2.7–97.3)	66.7 (41.2–85.6)	14.3 (0.8–58.0)	92.3 (62.1–99.6)
	Culture	100.0 (31.0–100.0)	76.5 (49.8–92.2)	42.9 (11.8–79.8)	100.0 (71.7–100)
Fungi	Smear	62.5 (25.9–89.8)	66.7 (35.4–88.7)	55.6 (22.7–84.7)	72.7 (39.3–92.7)
	Culture	57.1 (20.2–88.2)	61.5 (32.2–84.9)	44.4 (15.3–77.4)	72.7 (39.3–92.7)
	Histopathology	90.0 (54.1–99.5)	100.0 (65.6–100.0)	100.0 (62.9–100.0)	90.9 (57.1–99.5)
MTBC	Smear	100.0 (19.8–100.0)	88.9 (63.9–98.1)	50.0 (9.2–90.8)	100.0 (76.0–100.0)
	Histopathology	100.0 (31.0–100.0)	94.1 (69.2–99.7)	75.0 (21.9–98.7)	100.0 (75.9–100.0)

*PPV, positive predictive value; NPV, negative predictive value; CI: Confident Intervals*.

## Discussion

We reported a retrospective study of the application of mNGS in the diagnosis of infectious pathogens in lung biopsy tissues. The mNGS offered the advantage of a less biased pathogen detection methodology through direct sequencing of the sample's extracted DNA. In the current study, mNGS successfully identified the infectious pathogens in 15 out of 20 patients. It covered a wider range of fastidious and anaerobic pathogens than conventional culture method. In P15, two obligate anaerobic bacteria were identified. *F. nucleatum* and *P. intermedia* are often isolated from periodontal lesions associated with various forms of periodontal disease (Ximénez-Fyvie et al., 2000; Baek et al., 2018). Several studies reported the pathogenic potential of *P. intermedia* in the respiratory tract and demonstrated that extracellular toxins of *P. intermedia* are cytotoxic for human alveolar type II cells and neutrophils (Ulrich et al., 2010) . The presence of *P. intermedia* in the oral cavity or lower respiratory tract may be a risk factor for severe pneumococcal pneumonia (Nagaoka et al., 2014).

It is noteworthy that our research indicated that the application of mNGS improved the diagnosis of pulmonary invasive fungal infections. In the clinical microbiology laboratory, tissues were homogenized in a glass grinder, and used for smear and culture. The grind processing may affect the isolation of *Zygomycetes* (such as *Rhizopus* and *Mucor*). The mNGS identified more *Zygomycetes* than that by culture method. Four samples with negative culture results were indicated as *Rhizopus* or *Mucor* by mNGS. In another three samples, *Mucor* was identified by mNGS, which were absent in culture results. The PPV of mNGS relative to histopathological examination of fungi was high at 100.0%, with 90.0% sensitivity and 100.0% specificity in our study. The mNGS combined with smear analyses could be used as a routine diagnostic tool in invasive fungal infections, which can reduce the turn-around time and provide accurate identification of fungi species (Figure 3).

The mNGS could provide a wide range of organism and microbial profiles, which were difficult to interpret. The lung microbiome should be considered when interpreting mNGS results. The microbiome of the lung has been particularly difficult to characterize due to prior assumptions about the community composition of the lung, the diversity of pathogens causing diseases, and sampling concerns (Pragman et al., 2018). Several genera were proposed as possible core genera of the lung microbiome, including *Pseudomonas, Streptococcus, Prevotella, Fusobacterium, Haemophilus, Veillonella*, and *Porphyromonas* (Morris et al., 2013; Yu et al., 2016). However, samples of most lung microbiome studies were bronchoalveolar lavages rather than lung tissues. The composition of the microbial genera in the lung biopsies detected in our study showed some similarities with other lung microbiome studies, but many of these genera were found in relatively smaller proportions. In our study, we applied a new data management pipeline for the identification of pathogenic species from mNGS data. The relative abundance of 30% at the genus level and at least fifty unique reads could be used as thresholds to indicate the presence of potential pathogens and background lung microbiome. However, this should not apply to the MTBC, the common pathogen in pulmonary infections (Zumla et al., 2015). MTBC included *M. tuberculosis, M. canettii, M. africanum*, and *M. bovis* in our mNGS database. The genome sequence of the four *Mycobacterium* species showed high genomic similarity (Gutierrez et al., 2005), so there are few species-specific sequences. In P2 and P4, *M. canettii* contained more unique reads than other species in MTBC, but the number of unique reads to different species was not more than 5. Among the MTBC, *M. canettii* is a peculiar member specifically diagnosed in dozens of tuberculosis patients with reported contacts to the Horn of Africa (Aboubaker et al., 2015). *M. canettii* differs from the other members of the MTBC by processing a larger 4.48 ± 0.05 Mb mosaic genome and producing cordless and smooth-looking mycobacteria (Gutierrez et al., 2005). We speculated that the identification of specific species of MTBC may not be accurate, due to the high genomic similarity between different species of MTBC. Moreover, not all sequences in difference among the reference genomes can be used for species classification in MTBC. To avoid misunderstanding, we recommended reporting the results of MTBC, rather than specific species of MTBC. Based on our research, whenever the MTBC was detected by mNGS, pulmonary tuberculosis should be considered. The mNGS of lung biopsies yielded reads from bacteria from the oropharyngeal or skin flora, as well as viruses and yeasts that were not considered causes of pulmonary infections in immunocompetent patients. However, in immunocompromised patients, interpretation of the normal flora or the presence of environmental microbes should be guided by clinical manifestations and related laboratory examination results. Hence, from our mNGS data, we propose to compile an extensive list of pathogens for which the matching reads should be kept and which can be considered as potential pathogens in lung infections in future mNGS analyses. Meanwhile, combined with the results of conventional laboratory-based diagnostic methods, the interpretation of mNGS data could be improved.

This study had limitations, such as the small sample size, the depth of mNGS and the high amount of human DNA fragments in lung biopsy tissues. The lung biopsy tissues were collected by invasive procedures, so the sample size was less than other respiratory specimens. Identification of pathogens by mNGS in lung biopsy tissues was challenging and meaningful. The samples selected in our study included patients with different infectious pathogens and noninfectious etiology. Further, we will increase the sample size for verification the optimal thresholds for pathogens identification. Meanwhile, the clinical prospective study with patient follow-up and treatment would have to be conducted to ensure that mNGS can accurately characterize the lung microbiome of samples and highlight the presence of lung pathogens. In addition, the sequenced biopsies generated varied largely, from 0.7 to 69 million reads per sample (Supplemental Table S1). The P9 sample has only 0.7 million reads but contains high proportions of microbes, which means that the microbe-human ratio was relative high in the original sample. We believed that the low sequencing depth can reflect the real microbes' constitutions at this condition. All the samples were planned to have a depth about 20 million reads, but it was affect by a lot of factors, such as the DNA concentration of each sample, the accuracy of pooling operation, and the cyclization efficiency of the library. If the DNA concentration was too low, a larger volume was used for pooling, but it may still affect the reads counts. Standardized procedures need to be developed in the further study to eliminate or reduce biases between samples.

Although there are some limitations, our study firstly indicated that mNGS could offer an improved detection of pulmonary infectious pathogens (or absence) in lung biopsy tissues, with potential benefits in speed and sensitivity.

## Author contributions

HW conceived and designed the study. HL and HG analyzed the data. HM collected the related clinical information. HM, QW, SL, and HC conducted clinical work associated with the study. YL provided technical support. HL wrote the draft, and HW revised it. All authors approved the final version.

### Conflict of interest statement

The authors declare that the research was conducted in the absence of any commercial or financial relationships that could be construed as a potential conflict of interest.
