# Supplementary material for: Detection of Pulmonary Infectious Pathogens From Lung Biopsy Tissues by Metagenomic Next-Generation Sequencing
Source: Front Cell Infect Microbiol. 2018 Jun 25;8:205. doi: 10.3389/fcimb.2018.00205 (PMC6026637; doi:10.3389/fcimb.2018.00205)
Supplement: Supplementary file 1 [file Table_1.DOC]

Supplemental Table S1. Details of metagenomic sequencing data of 20 lung biopsy samples.

| Patient ID | No. of raw reads | No. of bacterial reads | No. of fungal reads |
| --- | --- | --- | --- |
| P1 | 24,451,057 | 80,639 | 94,183 |
| P2 | 15,505,309 | 1,366 | 521 |
| P3 | 30,057,472 | 1,233 | 1,158 |
| P4 | 3,893,042 | 35,620 | 107 |
| P5 | 11,420,438 | 160 | 8 |
| P6 | 28,083,806 | 14,244 | 117 |
| P7 | 12,273,762 | 2,102 | 2,902 |
| P8 | 18,356,221 | 1,592 | 878 |
| P9 | 694,269 | 14,639 | 945 |
| P10 | 39,206,408 | 15,105 | 1,204 |
| P11 | 25,925,878 | 157,804 | 1,939 |
| P12 | 13,834,799 | 1,168 | 609 |
| P13 | 13,517,492 | 7,375 | 1,593 |
| P14 | 16,533,215 | 1,188,450 | 10 |
| P15 | 69,403,268 | 738 | 67 |
| P16 | 11,554,093 | 2,161 | 582 |
| P17 | 11,670,348 | 452 | 566 |
| P18 | 19,732,770 | 390,913 | 7,120 |
| P19 | 22,344,495 | 255,465 | 83,908 |
| P20 | 14,791,970 | 8,155 | 74 |
